# Unraveling the gut microbiota's role in salt-sensitive hypertension: current evidences and future directions

**DOI:** 10.3389/fcvm.2024.1410623

**Published:** 2024-07-18

**Authors:** Li Wang, Jihong Hu

**Affiliations:** ^1^ Public Health School, Gansu University of Chinese Medicine, Lanzhou, China; ^2^ Teaching Experiment and Training Center, Gansu University of Chinese Medicine, Lanzhou, China; ^3^ Key Laboratory of Dunhuang Medicine, Ministry of Education, Gansu University of Chinese Medicine, Lanzhou, China

**Keywords:** gut microbiota, hypertension, salt sensitivity, TH17, SSH

## Abstract

The gut microbiota plays a pivotal role in both maintaining human health and in the pathogenesis of diseases. Recent studies have brought to light the significant correlation between gut microbiota and hypertension, particularly focusing on its role in the development and advancement of SSH, a subtype characterized by elevated blood pressure in response to high salt consumption. The complexity of SSH's etiology is notable, with dysbiosis of the gut microbiome identified as a crucial contributing factor. The gut microbiota participates in the occurrence and development of SSH by affecting the host's immune system, metabolic function, and neuromodulation. Investigations have demonstrated that the gut microbes regulate the development of SSH by regulating the TH17 axis and the activity of immune cells. Moreover, microbial metabolites, such as short-chain fatty acids, are implicated in blood pressure regulation and affect the development of SSH. There is evidence to show that the composition of the gut microbiome can be altered through prebiotic interventions so as to prevent and treat SSH. This review aims to concisely sum up the role of gut microbiota in SSH and to discuss pertinent therapeutic strategies and clinical implications, thereby providing a valuable reference for further research and clinical practice in this area.

## Graphical Abstract

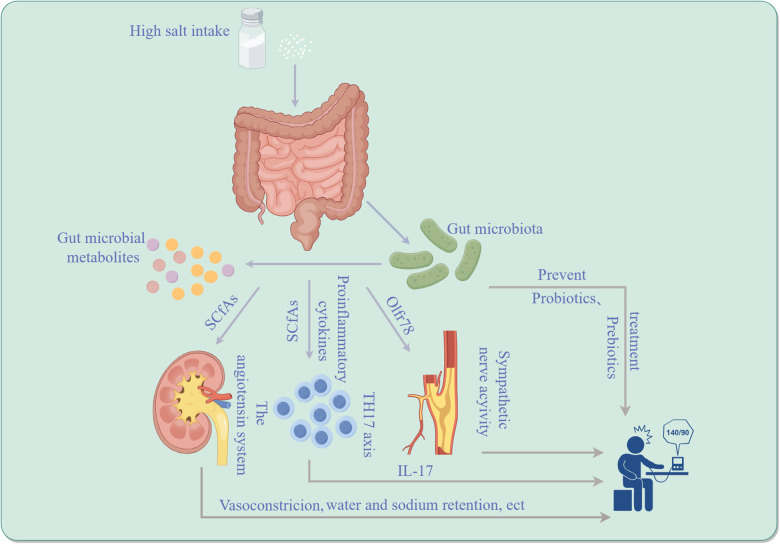

## 1 Introduction

High blood pressure is a world-wide health crisis, influencing 1.1 billion people and resulting in approximately 130 million deaths or severe complications such as stroke, heart disease, and kidney disease annually (1–3). Despite its complex genetic and environmental etiology, the pathogenesis of hypertension remains incompletely understood, leaving current treatments insufficient for all patients. High dietary salt intake is a key factor in raising blood pressure, as shown by various studies including epidemiological studies, animal research, and clinical trials (4, 5). Around half to three-quarters of people with high blood pressure are sensitive to salt, meaning their blood pressure rises abnormally when they consume too much salt. This is a significant risk factor for cardiovascular disease and mortality (6). Salt-sensitive hypertension (SSH) is linked to decreased salt excretion in the kidneys and causes a significant blood pressure response to changes in salt intake. High salt diet raises blood pressure significantly in SSH patients, while low salt diet lowers it. Conversely, the impact on blood pressure in non-salt-sensitive hypertension (NSSH) patients is less pronounced (7). SSH is believed to be caused by the kidneys not being able to properly excrete salt, leading to high levels of salt in the body. Research has shown that SSH is closely linked to genetics, neuroendocrine regulation, and abnormal immune system activation (8). For the past few years, a growing number of researches have shown that imbalanced gut microbes may also be an essential factor in SSH (9). The gut microbiota is a diverse community of microbes that is essential for the health of various organs and systems, including the gastrointestinal, immune, circulatory, and neural systems. Imbalances in the gut microbiota can impact not only intestinal health but also contribute to the development of hypertension by affecting blood pressure regulation (10). Excessive salt intake can disrupt intestinal flora balance and increase the risk of SSH (11). In addition, metabolic byproducts from gut microbes, such as short-chain fatty acids, can help regulate blood pressure by affecting blood vessel relaxation and the immune system (12). SSH is caused by the immune system, nervous system, endocrine system, and gut microbiota interacting (13). Further research is needed to fully understand the impact of gut flora on sodium-sensitive hypertension, but it is clear that studying the relationship between gut flora and hypertension is crucial for developing new prevention and treatment strategies. This review aims to comprehensively explore the involvement of intestinal flora in salt-induced high blood pressure, discussing treatment strategies and clinical implications, thereby offering new insights for its prevention and treatment.

## 2 Disturbance of intestinal microbiota and salt sensitivity of blood pressure

In the last few years, there has been growing interest in the role of gut microbes in both fitness and illness. Research has demonstrated a close association between intestinal microbial dysbiosis and the onset and progression of various chronic conditions, including hypertension (9). Meanwhile, gender plays a significant role in how gut microbiota and blood pressure salt sensitivity interact. Studies show that gender-specific genetic factors influence arterial stiffness and SSH differently (14). Gender may impact gut microbiota and SSH development, with gender-specific genetic factors influencing blood pressure regulation and other biological processes (14). Dysfunction of the proportion and function of the gut microbiota is called intestinal dysbiosis, which is also manifested as an excessive Firmicutes/Bacteroidetes ratio. This dysbiosis can lead to aberrant activation of the immune system and chronic low-grade inflammation, impacting host metabolism and blood pressure regulation. Studies indicate that an intestinal microbial imbalance in the gut can increase intestinal mucosal permeability, facilitating the invasion of bacteria and their byproducts into the circulatory system, thereby triggering systemic inflammatory responses and promoting hypertension (15). Yang, T. et al. (9) established a connection between gut microbiota and hypertension in animal models through bacterial DNA analysis from fecal samples of hypertensive rat models. This study revealed a substantial reduction in microbial variety, diversity, and uniformity, alongside an elevated Firmicutes to Bacteroidetes ratio in hypertensive rats compared to normal ones. Mell et al. (11) performed 16S rRNA gene sequencing on cecum samples from Dahl salt-sensitive and Dahl salt-resistant mice. They found an increased abundance of Bacteroidetes in rats that are sensitive to salt compared to salt-tolerant ones. Notably, when both rat types were subjected to a diet high in salt and given antibiotics to eliminate microbiota, transplantation of cecal contents from salt-sensitive or salt-resistant rats resulted in different blood pressure responses. Salt-sensitive rats transplanted with contents from salt-resistant rats exhibited sustained elevation in systolic blood pressure and a reduced lifespan compared to those transplanted with contents from salt-sensitive rats. These findings were corroborated by Yang et al.'s study (9), which consisted of a small cohort of 17 hypertensive patients, revealing a correlation between human hypertension and gut microbiota dysbiosis, with hypertensive patients exhibiting lower gut microbiota richness and diversity compared to controls. Furthermore, Yan (16) observed significant alterations in intestinal microorganisms and increased systolic and diastolic blood pressure in experimental rats fed a diet containing 8% sodium salt. Growing evidence underscores the pivotal role of gut microbes in SSH, potentially influencing the host's immune system, neuromodulation, and metabolic function. The gut microbiome profile in SSH patients differs from that in nonsalt-sensitive hypertensive patients, with specific species richness correlating with the severity of SSH (17). In addition to the aforementioned gut microbiota, Actinobacteria and Proteobacteria are also closely associated with SSH, as shown in Table 1.

**Table 1:** The gut microbiota may be related to SSH.

References	Phylum	Class	Order	Family	Genus
Wilck et al. (13)	Firmicutes, Proteobacteria, Bacteroidota,	Bacteroidia, Gammaproteobacteria	Lactobacillales, Enterobacteriales	Lactobacillaceae, Enterobacteriaceae, Prevotellaceae,	Lactobacillus, Escherichia coli, Oscillibacter, Clostridium XIVa, Johnsonella, Rothia, Parasutterella, Pseudoflavonifractor, Clostridia, Akkermansia, Alistipes
Chakraborty et al. (18)	Firmicutes, Bacteroidota, Actinobacteria	Bacteroidia, Actinobacteria	Bacteroidales, Lactobacillales, Clostridiales, Actinomycetales	Lachnospiraceae, Streptococcaceae, Sutterellacea Veillonellaceae, Clostridiaceae, Helicobacteriaceae, Erysipelotrichaceae, Ruminococcaceae, Actinomycetaceae	Lactobacillus, Sutterella, Prevotella, Paraprevotella, Turicibacter, Allobaculum, Anaerostipes
Miranda et al. (19)	Firmicutes	Clostridia	Clostridiales, Oscillospirales, Lactobacillales	Lachnospiraceae, Erysipelotrichaceae, Ruminococcaceae	Lactobacillus, Blautia, Bifidobacterium, Turicibacter, Oscillospira
Bier et al. (20)	Firmicutes	Clostridia	Pseudomonadales	Christensenellaceae, Barnesiellaceae, Eubacteriaceae	Erwinia, Anaerofustis, Anaerostipes
Abais-Battad et al. (21)	Firmicutes, Bacteroidota	Clostridia	Bacteroidales	Erysipelotrichaceae, Desulfovibrionaceae	Parabacteroides gordonii, Streptococcus alactolyticus,
Wang et al. (22)	Firmicutes, Bacteroidota, Verrucomicrobia, Actinobacteria, Proteobacteria	Bacteroidia, Actinobacteria	Bacteroidales, Lactobacillales, Enterobacteriales, Actinomycetales	Ruminococcaceae, Lachnospiraceae, Erysipelotrichaceae, Actinomycetaceae,	Ruminococcus, Lactobacillus, Bacteroidales S24-7, Alistipes, Lachnospiraceae NK4A136, Faecalibaculum, Bifidobacterium, Akkermansia, Rikenellaceae RC9, Ruminococcaceae UCG-014
Chen et al. (23)	Firmicutes, Bacteroidota, Proteobacteria	Bacteroidia, Deltaproteobacteria	^_^	Enterobacteriaceae, Sphaerochaeta, Ruminococcaceae, Rikenellaceae, Desulfovibrionaceae,	Lactobacillus, Proteobacteria, Alistipes, Wolbachia, Ruminiclostridium_6, Desulfovibrio, Anaeroplasma, Fusobacterium, Klebsiella, Sphaerochaeta, Ruminococcaceae UCG-014

^_^ , no relevant information provided in the studies.

## 3 Metabolites of the gut microbiota and blood pressure sensitivity to salt

Metabolic products of gut bacteria have been identified as significant factors in the regulation of salt-induced high blood pressure (11). For instance, metabolites, such as short-chain fatty acids (SCFAs) and aromatic compounds, can affect the angiotensin system, immune system, and neuromodulation, thereby impacting blood pressure levels (24). Short-chain fatty acids are derived from the gut microbiota fermentation of dietary fiber, such as resistant starch. The primary metabolites produced, including acetate, propionic acid, and butyrate, constitute 95% of SCFAs produced by the gut microbiota and regulate blood pressure by binding to specific receptors or by affecting the autonomic nervous system and immune system (25). G protein-coupled receptor 41 (GPR41) and olfactory receptor 78 (Olfr78) are widely expressed in sympathetic nervous ganglia, vascular endothelial cells, surface cells, juxtaglomerular complex and non-striated muscle cells, and play crucial responsibilities in modulating blood pressure through SCFAs. Researche has indicated that the activation of GPR41 can inhibit the differentiation of TH17 cells and the creation of inflammatory factors, thereby reducing vascular inflammation and protecting vascular function (26). In a SSH animal model, the down-regulation of GPR41 expression is significantly correlated with elevated blood pressure, implying that gut microbes may influence the development of SSH by regulating the activation state of GPR41 (26). In a population-based study (27), an increase in fecal butyrate was linked to lower systolic blood pressure among patients with cancer who were undergoing a weight-loss intervention. In another animal study (20), salt-sensitive rats were assigned to receive either a regular diet or a diet high in sodium. The concentrations of short-chain fatty acids in stools, such as acetic acid, propionic acid, butyric acid, and isobutyric acid, were determined by chromatography-mass spectrometry. The results suggest an interaction among a high-salt diet, gut microbial richness, and blood pressure in a mouse model of salt-exacerbated hypertension. Additionally, higher standards of SCfAs (ethanoic acid, propanoic acid, and isobutyric acid) were observed in bowel samples from hypertensive rats in the experimental group compared to the control group, and high salt disrupted SCFAs produced by the gut microbiota in salt-sensitive rats compared with those on a normal diet. Mell, B. et al. (11) quantified plasma SCFAs by mass spectrometry to investigate whether plasma SCFAs in salt-sensitive rats were altered by transplantation of cecal contents. Rats with salt sensitivity transplanted with cecal contents from resilient rats had higher plasma acetate and heptamate levels compared to rats prone to salt sensitivity transplanted with cecal contents from sensitive rats, suggesting that microbial composition influences SCFAs levels. Furthermore, A 6-week study showed that short-chain fatty acids increased on a low-sodium diet, including 2-methylbutyric acid, butyric acid, Isobutanoic acid, valeric acid, and caproic acid (28). Additionally, Exposure to LPS can increase salt sensitivity in the body, potentially leading to hypertension. This may be due to LPS-induced inflammation affecting blood pressure regulation and kidney function (29). Furthermore, tryptophan is important for SSH, with studies showing that its metabolites can lower blood pressure by affecting immune cells and gut microbiota (30–32). Studies have shown that bile acids and chlorogenic acid may impact SSH by regulating inflammation, fluid balance, and gut microbiota (33, 34).

## 4 Gut microbiota regulates the TH17 axis and SSH

There is a significant interaction between gut flora and the immune response. Modulating the composition and function of the gut microbiota may enhance the host's immune status, reduce inflammation, and subsequently lower blood pressure (35). The CD4^+^ T helper 17 (TH17) axis plays a significant role in regulating the immune system in SSH by influencing the activity of TH17 cells and the expression of Interleukin 17A (IL-17) through key factors like SGK1 and NFAT5, potentially contributing to the development of diseases like hypertension (36).

In experimental culture, T cells from salt-sensitive rats showed increased inflammation in high salt solution, indicating a combined effect of sodium chloride and cytokines in promoting TH17 cells and hypertension (37). Studies have unveiled the intricate interplay between the intestinal microbiota and the host's immune response, particularly its role in regulating SSH (38). In this process, specific immune cells, particularly T helper cells (TH cells), play a central role. TH17 cells, a type of T cell that produces the inflammatory cytokine IL-17, have been implicated in the onset of SSH (31). The composition and function of the intestinal flora can influence the polarization and function of TH17 cells through various mechanisms (13, 16, 39). For instance, substances generated by specific gut bacteria, such as short-chain fatty acids, can stimulate the generation of Treg cells, leading to the suppression of the activity of TH17 cells and reducing the risk of hypertension affected by salt. Conversely, an imbalanced gut microbiota may lead to excessive activation of TH17 cells and increased IL-17 fabrication, thereby promoting hypertension. Furthermore, the gut microbiome can indirectly regulate TH17 cells by impacting the integrity of the intestinal mucosal barrier (39, 40). A weakened intestinal barrier allows more outside antigens to enter the body, triggering the immune system and potentially worsening inflammation. This can impact the development of SSH by affecting the activity of TH17 cells through various mechanisms involving the gut flora (13, 38, 39). Extracellular vesicles (EVs) play a crucial role in SSH by transporting proteins, nucleic acids, and lipids among cells (41). Urinary EVs are being researched as possible biomarkers for hypertension, as they contain proteins related to renal sodium transport, which is important in hypertension development (42). Higher levels of NCC or phosphorylated NCC in urinary EVs are found in hypertensive patients, indicating the importance of EVs in hypertension. Other markers linked to hypertension, like Inducible Vitamin D Gene 2 protein and hsa-miR-4516, have also been identified in EVs, showing potential for early detection of hypertension (41–44). Thus, the gut microbiota may affect blood pressure in SSH by controlling salt absorption and metabolism through extracellular vesicles, potentially worsening the condition. Immune cells, T helper cells, and extracellular vesicles release pro-inflammatory cytokines, contributing to SSH. High salt intake leads to increased infiltration of specific cells, contributing to the development of this condition, as shown in Table 2.

**Table 2:** The immune cells may be related to SSH.

References	Immune cells	Class	Receptor molecules	Cytokines
Norlander et al. (39)	Adaptive immune cells	T lymphocytes	_	IL-6, IL-8, TNF-α, IL-17A
Dixon et al. (45)	Antigen-presenting cells, Adaptive immune cells	Dendritic cells(CD11c^+^, CD11b^−^, CD45RO^lo^, CD11c^hi^, CD11b^+^, CD45RO^hi^), B cells, Macrophages, T cells(CD8^+^)	CD86, CD80, CD70, CD27, CD83	TGF-*β*, IL-1β, IL-6, IL-23, IL-21, IL-17, IL-17A, IFN-*γ*, TNF-α, TGF-β1, GM-CSF, IL-17F,
Ferguson et al. (46)	Antigen-presenting cells, Adaptive immune cells	Dendritic cells(CD11c^+^), T cells(CD45^+^, CD3^+^, CD8^+^, CD4^+^), Monocyte/Macrophages(F4/80^+^) B cells(CD19^+^),	CD86	IFN-γ, IL-17A, IL-6, IL-17, IL-1β
Kambayashi and Laufer (47)	Antigen-presenting cells, Adaptive immune cells	Dendritic cells, T cells(CD4^+^)	CD80, CD86, CD4, CD40, CD40l	IL-4, IFN-γ, GM-CSF, IL-3, SCF, IL-9, IL-12, IL-12R
Saleem et al. (48)	Antigen-presenting cells, Adaptive immune cells	T cells(Th17, CD45^+^, CD3^+^, CD4^+^, CD8^+^,), Dendritic cells(CD11c^+^), B cell, Monocyte/Macrophages	CD14, Ly96, TLR4, CD28, CD80, CD86, CD4, CD8	IL-6, TNF-α, IL-1β, IL-17A, IL-18, IL-10, IFN-γ, IL-21
Lee et al. (49)	Adaptive immune cells	T cells(CD4^+^, Th17)	CD25, IL-23R	IL-17A, IL-10, IL-23
Ren and Crowley (50)	Antigen-presenting cells, Adaptive immune cells	T cells(CD4^+^, CD8^+^, Th17, CD25^+^) Dendritic cells	CD4, CD8, CD27, CD44, CD62, CD70, CD80, CD86, CD109, CD28	TNF-α, IFN-γ, IL-2, IL-4, IL-5, IL-9, IL-13, IL-17, IL-21, IL-22, IL-35, TGF-β1, IL-10, IL-17A, IL-6, IL-1β,TNF-α
Wilck et al. (13)	Adaptive immune cells	Dendritic cells, T cells(Th17, CD4^+^, RORγt^+^, CD25^+^, Foxp3^+^)	_	IL-17A, IFN-γ, TNF-α,

IL, interleukin; TNF, tumor necrosis factor; IFN, interferon; TGF, transforming growth factor; GM-CSF, granulocyte–macrophage colony-stimulating factor; ^_^, no relevant information provided in the studies.

## 5 The angiotensin system regulates gut microbiota metabolites and SSH

The angiotensin system constitutes a vital hormonal system in the human body primarily involved in regulating blood pressure and fluid balance (51, 52). Excessive salt consumption, particularly through a high-salt diet, activates the angiotensin system, initiating a series of physiological reactions. This system primarily functions through vasoconstriction, water and sodium retention, and sympathetic activation in the regulation of SSH (51, 53). Gut microbial metabolites are believed to influence the activity of the angiotensin system, thereby impacting the advancement and escalation of salt-induced high blood pressure. The relationship between the regulation of the angiotensin system (RAS) and the influence of intestinal microbe metabolites on SSH involves complex biological mechanisms. These mechanisms mainly encompass the direct effects of gut microbiota metabolites on RAS components and the effects of these metabolites on salt sensitivity regulation. Firstly, the gut microbiota can directly or indirectly affect angiotensin-converting enzyme (ACE) activity and angiotensin II (Ang II) production through its metabolites, particularly short-chain fatty acids such as butyric acid and propionic acid (40). SCFAs help lower blood pressure in SSH by affecting the adrenergic system and reducing sensitivity to salt response (54). Secondly, intestinal microflora indirectly affects the progression of hypertension sensitive to salt by influencing the control of the immune system. Gut microbial imbalance may lead to an increased systemic inflammatory response, exacerbating the pathological process of SSH by activating RAS and increasing Ang II levels. Conversely, a healthy gut microbiota may help inhibit this process and reduce SSH by producing anti-inflammatory compounds and modulating host immune responses (54). These discoveries emphasize the vital function of intestinal microbial metabolites in regulating RAS and SSH, offering potential strategies to treat and prevent SSH by adjusting the gut microbiota balance. Such strategies may involve promoting the growth of beneficial flora through diet, as well as the utilization of synbiotics and microbial adjuncts to affect RAS activity and reduce the risk of SSH (11, 54). Additionally, the angiotensin system has been associated with pathological processes, for instance, reactive oxygen species and immune response, which also play a significant function in the development of SSH (54).

## 6 Sympathetic nerve activity regulates the gut microbiota to affect compassionate neural network and SSH

The sympathetic nervous system, a component of the autonomic nervous system, primarily oversees the body's stress response and maintains internal stability (55). When the central nervous system detects excessive salt intake, it modulates the operation of the empathetic nerve structure through intricate neural pathways, potentially involving various brain regions such as the thalamus, amygdala, and brain stem (56). The interplay among these brain regions ultimately leads to heightened excitability of the sympathetic nervous system. This increased activity can impact the cardiovascular system, resulting in elevated cardiac rhythm, heightened myocardial contraction force, and increased peripheral vascular resistance (56). These physiological changes collectively elevate blood pressure, exacerbating the progression of sodium-reactive high blood pressure. Recent investigations have unveiled novel mechanisms through which the gut microbiota may influence renal renin secretion and blood pressure regulation via specific olfactory receptors (e.g., Olfr78) (57). The revelation that signaling substances generated by intestinal microbiota can act on these olfactory receptors to influence sympathetic nervous system activity offers a fresh understanding of the complex interaction between intestinal flora and systemic blood pressure control. This pathway shows how gut microbes can affect blood pressure and the development of SSH through communication with the olfactory receptor and sympathetic nervous system (15). Investigating how gut microbiota affects sympathetic nervous system activity may yield a deeper understanding of the mechanisms underpinning the evolution of sodium-reactive high blood pressure (15). Sodium-reactive high blood pressure patients exhibit abnormal sensitivity to salt intake, and the sympathetic nervous system participates in a central function in managing the body's hydration-sodium equilibrium and cardiovascular force. Consequently, the gut microbiota, through its influence on the sympathetic nervous system, may indirectly regulate salt absorption and blood pressure, thereby further impacting the development of SSH (15). This study not only elucidates a new mechanism through which gut microbiota may influence blood pressure regulation via the sympathetic nervous system but also lays the groundwork for the development of novel healing techniques for SSH (15). For instance, modifying the gut microbiota composition or targeting specific olfactory receptors may offer an effective means to modulate sympathetic activity, thereby controlling blood pressure and preventing SSH. In summary, gut microbiota influences sympathetic nervous system activity through specific olfactory receptors, with significant implications for the growth and advancement of sodium-responsive high blood pressure. This study not only advances our comprehension of the intricate connections between gut microbiota and blood pressure regulation but also points toward potential avenues for future hypertension treatments (15, 58). Elevated sympathetic activity is a key characteristic of SSH. Gut microbes can regulate blood pressure levels by influencing the host's nervous system activity. Gut microbes have been found to influence sympathetic nervous system activity, modulate renal saline balance, and regulate blood pressure (15). Specifically, gut microbial metabolites can affect the excitability and inhibition of the sympathetic nervous system through neurotransmission and hormone release pathways, thereby regulating blood pressure levels. This effect may be closely related to ward the advancement and evolution of sodium-reactive high blood pressure, particularly under conditions of a high-salt diet (58).

## 7 Gut microbiota prevents SSH

These studies indicate that the gut microbiota plays a crucial role in the development of SSH. Modulating the composition and metabolites of gut microbes can impact salt absorption and metabolism, thereby regulating blood pressure levels (59). Yang T. et al. (9) demonstrated that hypertension was linked to gut microbiota dysregulation in both creatures and people with high blood pressure. They suggested that nutritional treatment to adjust the intestinal flora might serve as a novel nutritional treatment strategy for hypertension. Yan, X et al. (16) revealed that a sodium-triggered rise in blood pressure could be reversed by administering the intestinal bacteria product linoleic acid, along with other known blood-pressure-lowering metabolites, such as short-chain fatty acids, produced by gut microbes fermenting prebiotic fibers. Probiotics and prebiotics, as nutritional supplements, have a certain effect on hypertension (60). Probiotics can reduce blood pressure levels by enhancing the composition and biochemical function of he intestinal flora, diminishing the number of harmful bacteria, and increasing the proportion of beneficial probiotics (61). Conversely, prebiotics supply nutrients to probiotics, promoting their growth and survival in the gut, thereby reinforcing the regulatory effect of probiotics on hypertension (62). Multiple studies in rats (63, 64) and a recent human study (65) have demonstrated that the intestinal microorganisms reduce blood pressure by fermenting brief-length oily chemicals generated from alimentary roughage. For instance, the absence of nutritional filament triggers high blood pressure in rodents, nevertheless; reintegration of SCFAs has shown a protective effect against hypertension and cardiac hypertrophy (60). Various approaches to modify the gut microbiota to ameliorate SSH have been explored, encompassing the utilization of friendly bacteria as well as prebiotics, as well as personalized strategies to modulate the gut microbiome (66). Among these, probiotics are considered a beneficial form of microbial supplementation, positively affecting blood pressure salt sensitivity by regulating the gut's internal environment, promoting the proliferation of beneficial bacteria, and enhancing metabolite production. Conversely, prebiotics are nutrients utilized by gut microbes and fermented to generate advantageous metabolites, such as SCFAs, that help maintain gut microbiota balance and diversity (65). Hence, utilizing gut microbiota to prevent and treat SSH represents an innovative research direction, offering new ideas for future personalized and precision medicine. Further investigation into the correlation between intestinal microorganisms and high blood pressure is expected to yield more effective treatment and treatment methods for patients with hypertension. By adjusting the composition composition of intestinal microorganisms, innovative approaches to prevent or manage salt-responsive high blood pressure may arise. Additional research is necessary to gain insight into the specific connections that are crucial in understanding various aspects of human health and disease, and how they collectively influence blood pressure regulation and the potential for high blood pressure.

## 8 Conclusion

New studies have demonstrated the crucial function of the intestinal microbiome in the prevention and treatment of SSH. Modulating the structure and substances of intestinal bacteria can impact salt absorption and metabolism, thereby regulating blood pressure levels. Research has suggested that dietary intervention to correct the gut microbiota could serve as a novel nutritional treatment strategy for hypertension. Additionally, administering gut microbial metabolites, such as arachidonic acid and short-chain fatty acids produced by gut microbes fermenting prebiotic fibers, has been found to reverse salt-induced increases in blood pressure. Probiotics and prebiotics have also shown promise in addressing hypertension. Probiotics lower blood pressure by improving gut bacteria, while prebiotics help probiotics grow and survive in the gut, strengthening their ability to regulate hypertension. Experiments in both animals and humans have shown that the intestinal microbiota can lower blood pressure by metabolizing SCFAs produced from dietary fiber. Reintroduction of short-chain fatty acids has shown a protective effect against hypertension and cardiac hypertrophy. Various approaches to modify the gut microbiota to ameliorate SSH have been explored, incorporating the application of probiotic supplements and prebiotics as well as personalized strategies to modulate the gut microbiome. Probiotics are considered a beneficial form of microbial supplementation, positively affecting blood pressure salt sensitivity by regulating the gut's internal environment, promoting the proliferation of beneficial bacteria, and enhancing metabolite production. Conversely, prebiotics are nutrients utilized by gut microbes, fermented to generate advantageous substances, like small-molecule fatty compounds, that aid in maintaining gut microbiota balance and diversity. Utilizing gut microbiota to prevent and treat SSH represents an innovative research direction, offering new ideas for future personalized and precision medicine. Further investigation into the connection between intestinal microorganisms and high blood pressure is expected to yield more effective treatment and treatment methods for patients with hypertension. Through modifying the structure of intestinal microorganisms, novel approaches for averting or managing salt-responsive high blood pressure could arise. Further research is needed to understand how gut bacteria and the immune system influence blood pressure regulation and susceptibility to hypertension.

## 9 Limitation and future directions

Research on how gut microbiota affects SSH has advanced, but challenges remain due to the use of animal models and the complexity of the gut microbiota. Understanding the multiple mechanisms involved in this relationship is still incomplete, and the diversity of gut microbiota in individuals complicates research efforts, necessitating more personalized approaches in future studies. Future research should prioritize conducting more human clinical trials to validate findings from animal model research and explore the mechanisms of interaction between gut microbiota and SSH. Additionally, further investigation is needed to understand how gut microbiota influences blood pressure regulation by affecting physiological systems like the sympathetic nervous system and the renin-angiotensin system, focusing on specific molecular and cellular mechanisms. Develop personalized treatment strategies, such as customized probiotics and prebiotics, to enhance prevention and treatment by considering individual variability in gut microbiota. Evaluate the long-term effects and safety of gut microbiota modulation for SSH prevention and treatment to support clinical application. In conclusion, the gut microbiota has potential for preventing and managing SSH. Further research is needed to understand its mechanisms and improve treatment strategies for patients with SSH.
